# Investigating the Molecular Mechanism of Xijiao Dihuang Decoction for the Treatment of SLE Based on Network Pharmacology and Molecular Docking Analysis

**DOI:** 10.1155/2022/5882346

**Published:** 2022-01-20

**Authors:** Fangzhi Wei, Yitian Song, Aiming Gong, Chengdan Pan, Yanping Zhuang, Xuan Zhang, Minyu Zeng

**Affiliations:** Department of Traditional Chinese Medicine, Hainan Medical University, Haikou 571199, China

## Abstract

**Objective:**

To elucidate the main mechanism of Xijiao Dihuang decoction (XJDHT) for the treatment of systemic lupus erythematosus (SLE).

**Methods:**

TCMSP, BATMAN-TCM, ETCM, and TCMID databases and literature search were used to screen the potential active compounds of XJDHT, and TCMSP and SwissProt databases were searched to predict the targets of the compounds. The targets of SLE were obtained from Genegards, OMIM, and DisGeNET databases, and Venn online platform was used to obtain the intersection targets of XJDHT and SLE. Afterwards, the PPI network was constructed by using the STRING database, and the core targets were identified by network topology analysis. GO and KEGG enrichment analyses were performed through R software, and molecular docking of the top three core targets and their corresponding compounds were accomplished by Autodock Vina and Pymol softwares.

**Results:**

There were 30 potential active ingredients, 289 potential targets, and 129 intersection targets screened from the above databases. Network topology analysis identified 23 core targets, such as AKT1, TNF, IL6, IL1B, and INS. GO enrichment analysis obtained 2555 terms and mainly clustering on the react to lipopolysaccharide, membrane raft, and ubiquitin-like protein ligase binding. KEGG enrichment analysis obtained 187 signaling pathways, mainly concentrating on the lipid and atherosclerosis, AGE-RAGE signaling pathway in diabetic complications, fluid shear stress, and atherosclerosis. Molecular docking verified that the active compounds of XJDHT have the strong binding activity to the core targets.

**Conclusion:**

This study preliminarily uncovers the mechanism of XJDHT acting on SLE through a “multicompound, multitarget, and multipathway” manner. XJDHT may achieve the treatment of SLE by inhibiting the proinflammatory factors, inflammatory signal cvtokines, proliferation, injury, and apoptosis processes. In summary, the present study would provide a promising theoretical basis for further clinical and experimental studies.

## 1. Introduction

SLE is a chronic autoimmune disease that involves multiple organs and tissues [1]. The incidence and prevalence of SLE are different around the world. The incidence in China is about 1/1000. In recent years, the prevalence of SLE has shown an upward trend [2]. The etiology and pathogenesis of SLE are not yet clear, which may be related to multiple factors such as genetic factors, environmental factors, and estrogen levels [3–5]. The treatment of SLE is still a difficult problem in the medical field, and modern medicine mostly uses glucocorticoids and immunosuppressive drugs [6], which may produce some side effects [7]. Traditional Chinese medicine (TCM) has rich experience in treating SLE with a wide variety of therapies. TCM not only improves the symptoms and the quality of survival of SLE patients but also reduces the side effects of glucocorticoid drugs and is cost-effective, thus it is a common treatment for SLE in Chinese clinics [8–10].

Xijiao Dihuang decoction (XJDHT) is from *Wai Tai Mi Yao*, compiled by Wang Tao in the Tang Dynasty. The formula consists of Bubali Cornu (Shuiniujiao, SNJ, 30 g), Paeoniae Radix Rubra (Chishao, CS, 10 g), Dried Rehmanniae Radix (Shengdihuang, SDH, 20 g), and Moutan Cortex (Mudanpi, MDP, 15 g), which exert the effects of clearing away heat and removing toxicity, cooling blood, and dispersing blood stasis. It is a basic prescription commonly used in TCM for the treatment of the syndrome of heat entering nutrient-blood. The clinical manifestations of SLE in the active stage mostly belong to the syndrome of exuberant heat and toxin, which coincides with the main therapeutic efficacy of XJDHT. However, the potential pharmacological mechanism of XJDHT against SLE is still unclear. The research model of “one drug-one target-one disease” cannot reflect the characteristics of TCM (multitarget and multipathway) [11].

**Figure 1:**
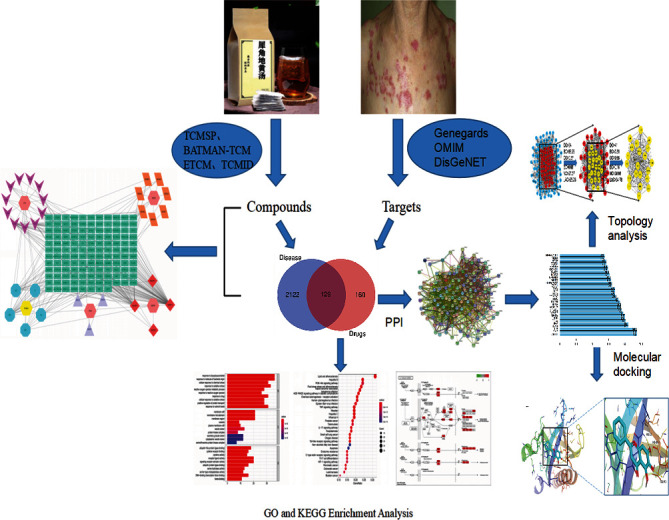
The flowchart of the analysis procedures of the study.

Network pharmacology and molecular docking provide a more complete understanding of network theory and systems biology, which also help to explain the mechanism of drugs [12]. In this study, we intend to use network pharmacology to screen the active ingredients and the intersection targets of XJDHT and SLE through data analysis and bioinformatics theory. The molecular docking technique was used to fit the binding activity between the active ingredients and the core targets to provide a theoretical basis for the key mechanism of XJDHT acting on SLE. The simplified flowchart of our study is shown in Figure 1.

## 2. Materials and Methods

### 2.1. Collection of Potential Active Ingredients and Related Targets of XJDHT

Before conducting the screening, we found that the main ingredients of the same Chinese medicine differed in different TCM databases, and the targets of the same compounds were inconsistent in different TCM databases. Each TCM database has its advantage and disadvantage. For instance, Traditional Chinese Medicine Database and Analysis Platform (TCMSP) database [13] (https://www.tcmsp-e.com/) is the only one that provides pharmacokinetic properties, such as oral bioavailability (OB) and drug likeness (DL), whereas it only collects 499 Chinese medicines. Bioinformatics Analysis Tool for Molecular mechANism of Traditional Chinese Medicine (BATMAN-TCM) database [14] (http://bionet.ncpsb.org.cn/batman-tcm/) is an online bioinformatics analysis tool that contains multiple functions, but it only offers relatively few Chinese medicine ingredients. The Encyclopedia of Traditional Chinese Medicine (ETCM) database [15] (http://www.tcmip.cn/ETCM/index.php/) includes standardized information for the commonly Chinese medicines and formulas of TCM; nonetheless, only 402 herbs have been collected. Traditional Chinese Medicines Integrated Database (TCMID) database (http://www.megabionet.org/tcmid/) contains 8159 kinds of Chinese medicines, which has the largest number of Chinese medicines in all databases, and each Chinese medicine ingredient retrieved is supported by relevant literature. However, it contains too little information on the targets of Chinese medicine ingredients. Therefore, we optimized the retrieval strategy. First of all, the ingredients of each Chinese medicine were retrieved from each of the four TCM databases mentioned above to obtain the main ingredients of XJDHT. Then, all the ingredients were initially screened in the TCMSP database according to OB ≥ 30% and DL ≥ 0.18. For the compounds that cannot be available in the TCMSP database, the compounds with high gastrointestinal absorption and good DL were further screened out by the SwissADME database (http://www.swissadme.ch/) [15]. In addition, we found that certain compounds, although with OB < 30% or DL < 0.18, have a wide range of pharmacological activities (such as oleic acid [16] and gamma-aminobutyric acid [17]) or are major ingredients of a certain Chinese medicine (such as jioglutin [18] and aspartic acid [19]), which had also been added as potential active ingredients of XJDHT. TCMSP database was used to forecast the relevant targets for potential active ingredients of XJDHT, and the names of the relevant targets were normalized through the UniProt database [20] (https://www.uniprot.org/). If TCMSP database cannot search for the compounds, the targets of the compounds would be predicted by Swiss Target Prediction (http://swisstargetprediction.ch/) to obtain all the potential targets of XJDHT, and the probability ≥ 0.12 was used as the screening condition.

### 2.2. Acquisition of Known Targets for SLE and Construction of Venn Diagrams

“Systemic lupus erythematosus” was used as a keyword to screen in GeneCards database (http://www.genecards.org/), OMIM database (https://omim.org/), and DisGeNET database (https://www.disgenet.org/). Meanwhile, Genecards database and DisGeNET database selected scores greater than 4 and 0.1, respectively, and all known targets were merged and removed duplicate values to obtain the union targets of SLE. Furthermore, we used Venn 2.1.0 online platform (http://bioinfogp.cnb.csic.es/tools/venny) to obtain the intersection targets of XJDHT against SLE.

### 2.3. Construction and Analysis of the Network of “Chinese Medicines-Active Ingredients-Intersection Targets”

Cytoscape 3.8.2 is a visualization software that can show the interactions and connections between things [21]. The active ingredients of XJDHT against SLE were obtained by searching the compounds corresponding to the intersection targets. Then, we imported Chinese medicines, active ingredients, and intersection targets into Cytoscape 3.8.2 software to construct the network diagram of “Chinese medicines-active ingredients-intersection targets.” The nodes of the network diagram represent Chinese medicines, active ingredients, and intersection targets, and the edges represent their interactions. The core active ingredients in the network were analyzed according to the degree values of the ingredients.

### 2.4. Construction and Analysis of PPI Network and Core Network

STRING (http://string-db.org/) is a database containing the known and predicted protein-protein interactions (PPIs) [22]. The intersection targets of XJDHT and SLE were uploaded to the STRING online database platform to obtain the PPI network diagram of the intersection targets by setting the organism as “Homo sapiens” and the minimum required interaction scores ≥ 0.4. Afterwards, the PPI network diagram and tsv format file were exported. The network topology analysis of the parameters in the PPI network was performed with the help of R 4.0.5 software and CytoNCA plug-in [23] of Cytoscape 3.8.2 software to finally obtain the core targets of XJDHT against SLE.

### 2.5. GO and KEGG Pathway Enrichment Analysis

Firstly, we installed the relevant R packages such as “BiocManager,” “cluster Profiler,” and “pathview” [24] to perform Gene Ontology (GO) and Kyoto Encyclopedia of Genes and Genomes (KEGG) pathway enrichment analysis, and the results were visually displayed through R 4.0.5 software. Meanwhile, GO analysis includes three aspects of molecular function (MF), biological process (BP), and cellular component (CC). Besides, the statistical significance of enrichment analysis was *P* ≤ 0.05. Finally, the “Pathview” package was used to map the signaling pathway containing the most intersection targets.

### 2.6. Molecular Docking Prediction

The top three core targets in the PPI network and their corresponding compounds were selected as the receptors and the ligands for molecular docking, respectively. The crystal structure of the receptor proteins was retrieved from the RCSB PDB database (https://www.rcsb.org/), and the water molecules of the receptor proteins were removed by PyMOL 2.4.1 software. Then, the optimized receptors were imported into Auto Dock tools1.5.6 software for hydrogenation and calculation of charge, and the output results were saved as pdbqt format. The ligand 2D structures were downloaded from the PubChem database (https://pubchem.ncbi.nlm.nih.gov/) and imported into the ChemBio3D Ultra 17.0 software for 3D structure conversion and optimization of the mechanical structure. The 3D structures of the ligands were saved as mol2 format files and imported into AutoDock Tools 1.5.6 software to save as pdbqt format. Molecular docking was performed by AutoDock vina 1.1.2 software [25], and the results were analyzed and visualized by PyMOL 2.4.1 software [26].

## 3. Results

### 3.1. Screening of Potential Active Ingredients and Targets of XJDHT

Through retrieving TCMSP, BATMAN-TCM, ETCM, and TCMID databases and related literature, we finally obtained 60 potential active ingredients. Although 13 of these ingredients fail to meet the screening criteria of OB ≥ 30% or DL ≥ 0.18, they either had a broad pharmacological activity or widely existed in multiple drugs of XJDHT, which were also considered as potential pharmacological active ingredients. The potential active ingredients of SNJ, CS, SDH, and MDP were 6, 32, 10, and 12, respectively, and the basic information of the potential active ingredients of XJDHT is shown in Table 1. The TCMSP and Swiss Target Prediction databases were searched to acquire 693 targets for the potential active ingredients, but only 625 targets were available to exclude some targets, which were without corresponding gene names or not acting on humans. The numbers of potential targets for SNJ, CS, SDH, and MDP were 98, 199, 244 and 84, respectively. Eventually, 289 valid targets were identified after removing duplicate values.

### 3.2. Acquisition of XJDHT and SLE Intersection Targets

**Figure 2:**
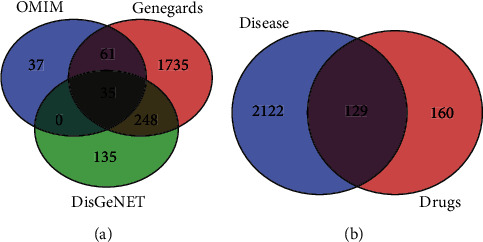
Venn diagrams. (a) Venn diagram of SLE-related genes. (b) Venn diagram of XJDHT targets and SLE targets.

By retrieving the disease database, we obtained 133, 2079, and 416 known targets from the OMIM database, Genecards database, and DisGeNET database, respectively. A total of 2251 known targets of SLE targets were obtained after taking union and removing duplicate values. The potential targets of XJDHT were mapped to the union targets of SLE, and 129 intersection targets were available (Figure 2).

### 3.3. Construction and Analysis of the Network of “Chinese Medicines-Active Ingredients-Intersection Targets”

**Figure 3:**
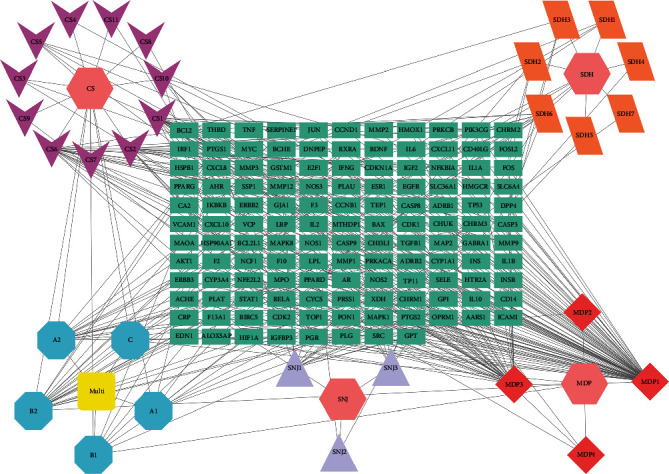
Chinese medicines-active ingredients-intersection targets network of SLE. The hexagon nodes represent all the herbs of XJDHT, the V nodes represent Paeoniae Radix Rubra, the diamond nodes represent Moutan Cortex, the parallelogram nodes represent Dried Rehmanniae Radix, the triangle nodes represent Bubali Cornu, the rectangle nodes represent the intersection genes, the octagon nodes represent compounds derived from multiple drugs, and the round rectangle node represents multiple compounds.

A total of 30 active ingredients of XJDHT for the treatment of SLE were obtained by precisely matching the compounds corresponding to the intersection targets. “Chinese medicines-active ingredients-intersection targets” network was constructed by Cytoscape 3.8.2 software (Figure 3), which included 164 nodes and 339 edges. The degree value of a node indicates the number of nodes in the network that interact directly with the node, and the higher the degree value of a compound, the more important it is in the network. According to the results of a topological analysis, the top 6 core compounds were MDP1 (quercetin), MDP3 (kaempferol), CS6 (beta-sitosterol), CS1 (baicalein), B2 (Stigmasterol), and A2 (oleic acid) in the network, which indicated that the above core active compounds play an important role in the treatment of SLE.

### 3.4. Construction and Analysis of PPI and Core Targets Network

**Figure 4:**
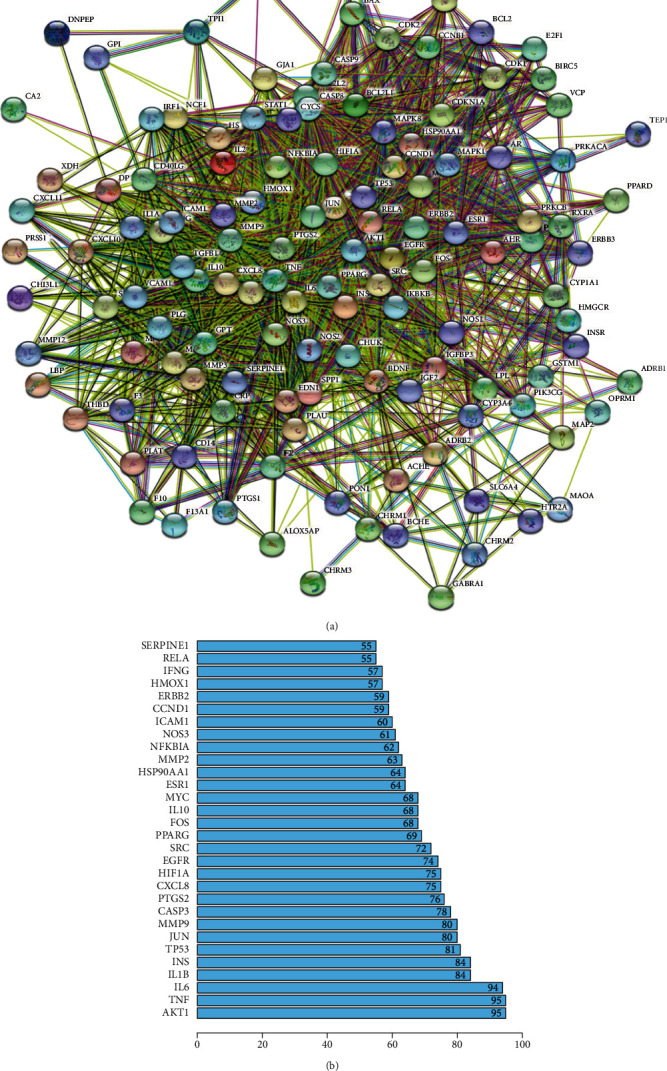
(a) PPI network of the intersection targets of XJDHT against SLE. (b) The top 20 significant genes in PPI network.

**Figure 5:**
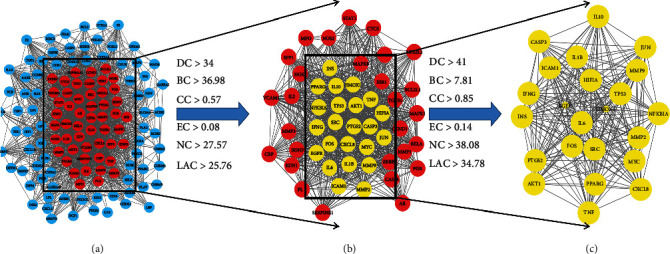
The network topology analysis of the PPI network. (a) The PPI network of the intersection targets was generated by Cytoscape, which is comprised of 124 nodes and 2324 edges. (b) The subnetwork after DC, BC, CC, EC, NC, and LAC filtration, which is comprised of 51 nodes and 1044 edges. (c) The core network after DC, BC, CC, EC, NC, and LAC filtration, which is comprised of 23 nodes and 252 edges.

The intersection targets were used to construct the PPI network by STRING online database platform (Figure 4(a)). A total of 127 nodes (with 2 disconnected nodes removed) and 2324 edges were obtained, and the top 20 targets of the degree value in the PPI network were plotted as bars by R 4.0.5 software (Figure 4(b)). The core targets were screened by the CytoNCA plugin of Cytoscape 3.8.2, and the subnetwork of 23 core targets was obtained by twice filtering with scores of betweenness centrality (BC), closeness centrality (CC), degree centrality (DC), eigenvector centrality (EC), local average connectivity-based method centrality (LAC) and network centrality (NC) higher than the median value (Figure 5). Comparing the core targets of Figures 3 and 4, we discovered that the core targets in both figures were basically consistent. The top 10 targets in both figures were RAC-alpha serine/threonine-protein kinase (AKT1), tumor necrosis factor (TNF), interleukin-6 (IL6), interleukin-1 beta (IL1B), insulin (INS), cellular tumor antigen p53 (TP53), transcription factor AP-1(JUN), matrix metalloproteinase-9 (MMP9), caspase-3 (CASP3), and prostaglandin G/H synthase 2 (PTGS2), which suggested that these targets were the core targets of XJDHT for the treatment of SLE.

### 3.5. GO and KEGG Enrichment Analysis

**Figure 6:**
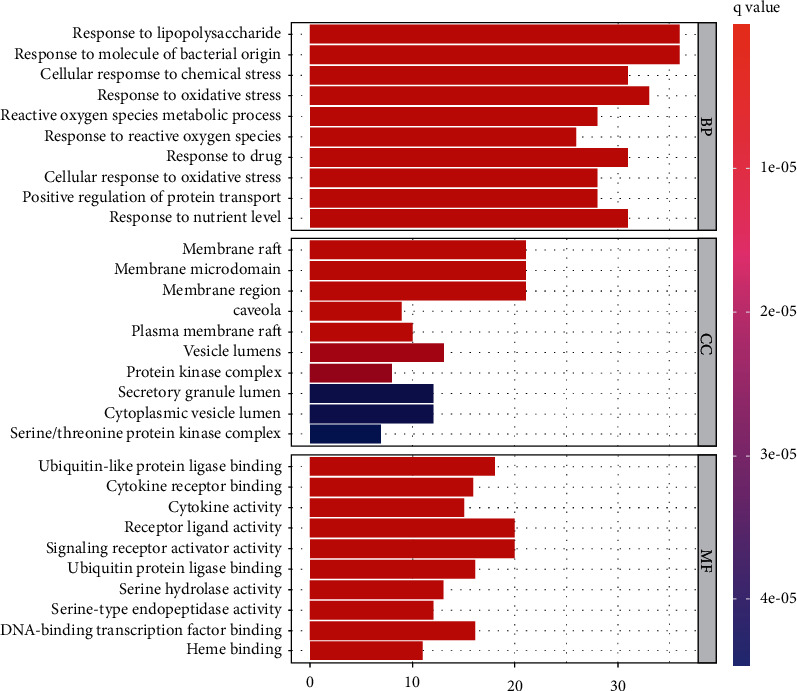
Gene Ontology terms of 129 intersection targets. The top 10 GO functional terms were selected (*P* ≤ 0.05). Abbreviations: BP: biological processes; CC: cellular component; MF: molecular function.

**Figure 7:**
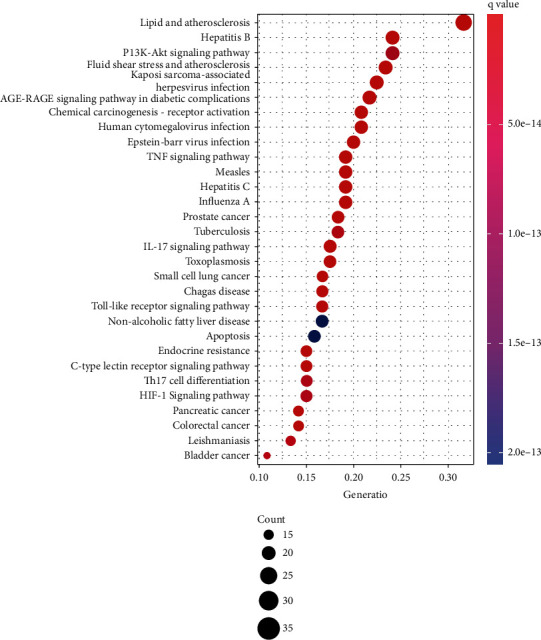
KEGG pathway enrichment of 129 intersection targets. The top 30 pathways were identified. Color represented *P* value, and size of the spot represented count of genes.

**Figure 8:**
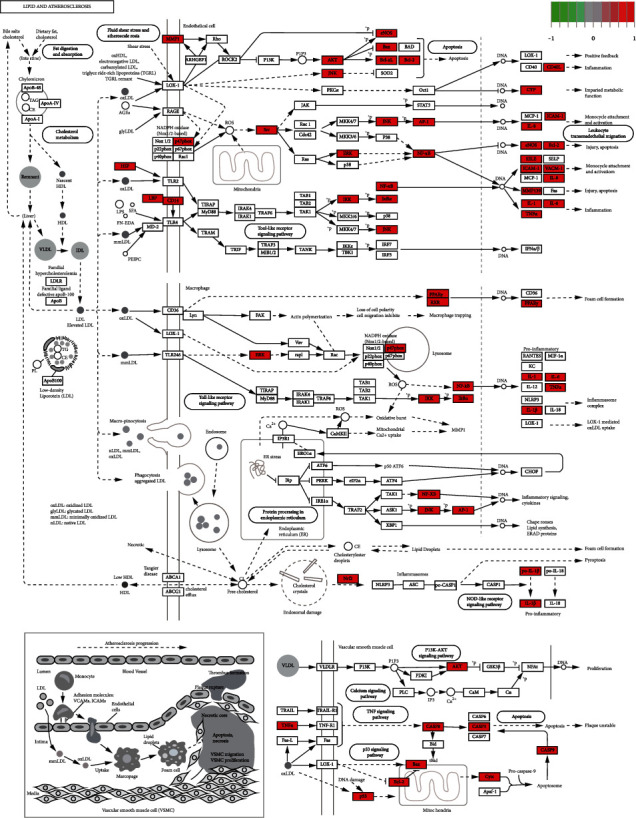
Lipid and atherosclerosis signaling pathway. Red represents the intersection targets of XJDHT against SLE in the signaling pathway.

GO and KEGG enrichment analysis were performed through R 4.0.5 software, and 2535 GO terms were identified by GO enrichment analysis. The top 10 representative clusters of BP, CC, and MF were screened according to Log*P* values (Figure 6). Through the pictures, we can find that the top three BP were the respond to lipopolysaccharide, respond to molecule of bacterial origin, and respond to cellular response to chemical stress, and the top three CC were the membrane raft, membrane microdomain, and membrane region, and the top three MF were the ubiquitin-like protein ligase binding, cytokine receptor binding, and cytokine activity. KEGG enrichment analysis screened out 178 signaling pathways, which were mainly enriched in the lipid and atherosclerosis, AGE-RAGE signaling pathway in diabetic complications, fluid shear stress and atherosclerosis, hepatitis B, prostate cancer, TNF signaling pathway, and IL-17 signaling pathway. Afterwards, we selected the top 30 signaling pathways with the highest significance for the visual presentation (Figure 7). Finally, the signaling pathway with the most intersection targets was plotted (Figure 8), which contained a total of 38 intersection targets.

### 3.6. Validation of Molecular Docking

**Figure 9:**
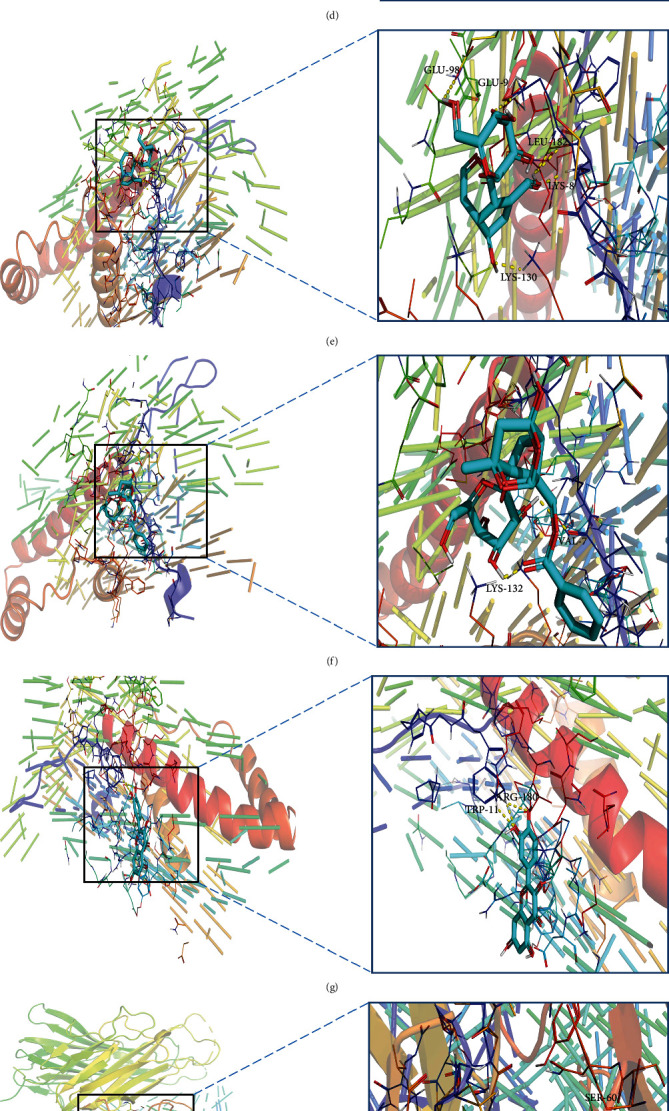
Molecular dockings of the top 3 core targets and their corresponding compounds. (a) AKT1 and baicalein. (b) AKT1 and kaempferol. (c) AKT1 and quercetin. (d) IL6 and gamma-aminobutyric acid. (e) IL6 and acubin. (f) IL6 and paeoniflorin. (g) IL6 and quercetin. (h) TNF and aucubin. (i) TNF and kaempferol. (j) TNF and paeoniflorin. (k) TNF and quercetin.

The lower the intermolecular binding energy, the better the docking effect. The binding energy ≤ −5 kcal/mol generally indicates that the receptors and ligands have relatively good binding properties [27]. To further investigate the binding activity of the main active compounds of XJDHT and the top 3 core targets in the PPI network and their corresponding compounds was performed as molecular dockings, respectively (Table 2 and Figure 9). The binding energies of the core targets and the corresponding compounds were almost ≤-5 kcal/mol, which indicated that the affinity of the core targets and their corresponding compounds were generally high. From Table 2, we found that Paeoniflorin and TNF had the best binding ability, with the lowest binding energy = −13 kcal/mol. Through molecular docking verification, we can conclude that the core targets of XJDHT acting on SLE and the corresponding active ingredients have certain or even strong binding ability, which verifies the credibility of the network pharmacological results.

## 4. Discussion

SLE is a classic autoimmune disease that severely affects the quality of life of patients. If not treated adequately, it can accelerate multiorgan and multitissue damage. The clinical efficacy of TCM against SLE is effective, not only can it improve the symptoms of SLE patients but also is affordable for the average patients. XJDHT is a fundamental formula for the treatment of SLE with the syndrome of exuberant heat and toxin, which can alleviate the clinical symptoms of SLE patients such as butterfly-shaped erythema of the face, purple spots on the skin, high fever, irritability, and joint and muscle pain, but the molecular mechanism of action is still unclear. Related studies have proved that XJDHT can regulate inflammation, protect nerves, and inhibit apoptosis, which may treat SLE through these effects [28, 29].

By analyzing the “Chinese medicines-active ingredients-intersection targets” network, we found that the core active compounds in XJDHT were quercetin, kaempferol, beta-sitosterol, baicalein, and stigmasterol. Quercetin is a natural flavonoid with anti-inflammatory, antioxidant, immunomodulatory, and neuroprotective properties [30], which can inhibit CD4 T cell activation and anti-inflammatory effects of macrophages to improve the symptoms in lupus nephritis (LN) mice [31]. Dos Santos et al. [32] observed that quercetin produced nephroprotective effects in LN mice through decreasing proteinuria levels and tissue expression of IL-6 and TNF-*α*. Liu et al. [33] identified that quercetin inhibited mesangial cell overproliferation in LN mice by suppressing the activation of NF-*κ*B signaling pathway and decreasing PTX3 expression. Kaempferol can enhance the suppressive function of regulatory T cells (Tregs) by reducing PIM1-mediated FOXP3 phosphorylation at S422, thereby preventing and treating SLE [34]. Macrophages are closely related to the pathogenesis of SLE [35–37]. Beta-sitosterol can regulate macrophage function [38]; therefore it may treat SLE by regulating macrophage function. Baicalin can adjust the balance of Nrf2/HO-1 signaling and NLRP3 expression in myeloid-derived suppressor cells (MDSCs) and reduce proteinuria and renal impairment in LN mice [39].

PPI network analysis identified AKT1, TNF, IL6, IL1B, INS, and TP53 as the core targets of XJDHT for the treatment of SLE. AKT1 is a serine-threonine protein kinase, which participates in various biological processes such as metabolism, cell survival, insulin signaling, and angiogenesis [40]. Increased AKT1 gene expression is associated with T-helper-transcription factors in SLE patients [41]. The pathogenesis of SLE is related to the activation of AKT/mTOR pathway by AKT1 downregulation of miR-633 [42]. TNF is a cytokine secreted by macrophages and an immunomodulatory molecule that can alter the balance of T-regulatory cells and participate in the pathogenesis of SLE [43]. Several studies have shown that TNF-*α* gene polymorphisms are closely related to the susceptibility to SLE [44–46]. IL6 is a cytokine with multiple biological functions in immunity and tissue regeneration, which is associated with the pathogenesis of SLE [47]. Ruchakorn et al. [48] suggested that IL-6 is related to the risk of active nonrenal SLE. Shaltout et al. [49] verified that IL6 may play a role in SLE pathogenesis through effecting on double negative T cells and anti-ds-DNA. IL1B is a potent proinflammatory cytokine that promotes Th17 differentiation of T cells, and its ratio in SLE patients with disease activity is less than in SLE patients with moderate disease [50]. TP53 is a central regulator of apoptosis [51], and its rs1042522G/C polymorphism is significantly associated with SLE in Chinese Han population [52]. Teng et al. [53] revealed that the high expression of miR-564 in patients with SLE promoted differentiation of dendritic cells by negatively regulating TP53 expression.

Conducting GO analysis on the intersection genes, we found that XJDHT exerted its effects on SLE through the response to lipopolysaccharide, response to oxidative stress, cytokine receptor binding, and cytokine activity. KEGG enrichment analysis demonstrated that the main pathways involve in the lipid and atherosclerosis, AGE-RAGE signaling pathway in diabetic complications, and fluid shear stress and atherosclerosis, and each of the each pathway contains multiple targets. The number of intersection targets of the pathway indicates the importance of the pathway. The pathway with the highest number of genes was the lipid and atherosclerosis, which contains 38 targets. It mainly involves phosphoinositide 3-kinase- (PI3K-) AKT, TNF, c-Jun N-terminal kinase (JNK), and mitogen-activated protein kinase 1/3 (ERK) pathway, and their functions are mainly focused on the proinflammatory, inflammatory signal cvtokines, proliferation, injury, and apoptosis processes. PI3K-AKT signaling pathway plays an important role in cellular proliferation, and AKT expression is increased in SLE patients [54]. Dysregulation of the PI3K-AKT pathway in the MRL/lpr lupus mice was particularly prominent [55]. Furthermore, upregulation of FoxO3a expression by PI3K-AKT pathway attenuated the progression of nephritis in LN mice [56]. JNK and ERK often interact with each other and are both associated with apoptosis [57], and their activity is positively correlated with SLE activity [58]. The reduction of ERK/JNK ratio can predict the severity of organ damage in SLE patients [59].

Molecular docking results demonstrated that the docking energies of the core targets and their corresponding active ingredients were almost all less than -5 kcal/mol, which indicates that the active ingredients of XJDHT have the good binding activity to the core targets.

In this study, the molecular mechanism of XJDHT for the treatment of SLE was elucidated through the screening of the active ingredients and the enrichment analysis of the intersection targets. The active ingredients of XJDHT (such as quercetin and kaempferol) act on the core targets (like AKT1 and TNF), which affect multiple signaling pathways to treat SLE. Molecular docking initially validated the interaction patterns of the major active ingredients with the core targets. However, there are still some limitations. The present study lacks the consideration of the interactions between the ingredients and the content of each ingredient. In addition, the results should be undertaken by further experiments.

## 5. Conclusions

In conclusion, this study preliminarily revealed the pharmacological effects of XJDHT against SLE through network pharmacology and molecular docking methods. A total of 30 active ingredients from XJDHT were discovered to be associated with SLE, and 23 corresponding genes were identified as core targets. The molecular mechanism of XJDHT acting on SLE is intimately related to the key GO terms and KEGG signaling pathways, such as the react to lipopolysaccharide, membrane raft, ubiquitin-like protein ligase binding, lipid, and atherosclerosis, AGE-RAGE signaling pathway in diabetic complications, fluid shear stress, and atherosclerosis. XJDHT may achieve the treatment for SLE by inhibiting the proinflammatory factors, inflammatory signal cvtokines, proliferation, injury, and apoptosis processes. This study provides the foundation for further research on the mechanism of XJDHT in the treatment of SLE.

## Figures and Tables

**Table 1:** All the potential pharmacologically active ingredients of XJDHT.

Herb name	Molecule ID	Molecule name	OB(%)	DL
Bubali Cornu (Shuiniujiao, SNJ)	MOL000987	Cholesterol	37.87	0.68
	MOL000054	Arginine	47.64	0.03
	MOL000065	Aspartic acid	79.74	0.02
	MOL000042	Alanine	87.69	0.01
	MOL001443	4-Guanidino-1-butanol	26.23	0.01
	MOL006394	Guanidine	24	0
Paeoniae Radix Rubra (Chishao, CS)	MOL001921	Lactiflorin	49.12	0.80
	MOL001924	Paeoniflorin	53.87	0.79
	MOL007004	Albiflorin	30.25	0.77
	MOL000449	Stigmasterol	43.83	0.76
	MOL004355	Spinasterol	42.98	0.76
	MOL002776	Baicalin	40.12	0.75
	MOL000358	Beta-sitosterol	36.91	0.75
	MOL000359	Sitosterol	36.91	0.75
	MOL006999	Stigmast-7-en-3-ol	37.42	0.75
	MOL005043	Campest-5-en-3beta-ol	37.58	0.71
	MOL007025	Isobenzoylpaeoniflorin	31.14	0.54
	MOL007003	Benzoyl paeoniflorin	31.14	0.54
	MOL007014	8-Debenzoylpaeonidanin	31.74	0.45
	MOL007008	4-Ethyl-paeoniflorin_qt	56.87	0.44
	MOL001002	Ellagic acid	43.06	0.43
	MOL007012	4-O-methyl-paeoniflorin_qt	56.70	0.43
	MOL001925	Paeoniflorin_qt	68.18	0.40
	MOL007016	Paeoniflorigenone	65.33	0.37
	MOL001918	Paeoniflorgenone	87.59	0.37
	MOL006996	1-O-beta-d-glucopyranosylpaeonisuffrone_qt	65.08	0.35
	MOL007005	Albiflorin_qt	48.70	0.33
	MOL006992	(2R,3R)-4-methoxyl-distylin	59.98	0.30
	MOL006994	1-O-beta-d-glucopyranosyl-8-o-benzoylpaeonisuffrone_qt	36.01	0.30
	MOL007018	9-Ethyl-neo-paeoniaflorin A_qt	64.42	0.30
	MOL006990	(1S,2S,4R)-trans-2-hydroxy-1,8-cineole-B-D-glucopyranoside	30.25	0.27
	MOL000492	(+)-catechin	54.83	0.24
	MOL007022	EvofolinB	64.74	0.22
	MOL002714	Baicalein	33.52	0.21
	MOL002883	Ethyl oleate (NF)	32.40	0.19
	MOL000131	EIC	41.9	0.14
	MOL000675	Oleic acid	33.13	0.14
	MOL001746	ELD	31.20	0.14
Moutan Cortex (Mudanpi, MDP)	MOL000211	Mairin	55.38	0.78
	MOL000359	Sitosterol	36.91	0.75
	MOL007003	Benzoyl paeoniflorin	31.14	0.54
	MOL007369	4-O-methylpaeoniflorin_qt	67.24	0.43
	MOL001925	Paeoniflorin_qt	68.18	0.40
	MOL007382	Mudanpioside-h_qt 2	42.36	0.37
	MOL007384	Paeonidanin_qt	65.31	0.35
	MOL007374	5-[[5-(4-methoxyphenyl)-2-furyl]methylene]barbituric acid	43.44	0.30
	MOL000098	Quercetin	46.43	0.28
	MOL000492	(+)-catechin	54.83	0.24
	MOL000422	Kaempferol	41.88	0.24
	MOL000675	Oleic acid	33.13	0.14
Dried Rehmanniae Radix (Shengdihuang, DH)	MOL000449	Stigmasterol	43.83	0.76
	MOL000359	Sitosterol	36.91	0.75
	MOL012254	Campesterol	37.58	0.71
	MOL002819	Catalpol	5.07	0.44
	MOL003735	Aucubin	4.17	0.33
	MOL000842	Sucrose	7.17	0.23
	MOL000131	EIC	41.9	0.14
	MOL003708	Jioglutin D	39.02	0.14
	MOL003706	Jioglutin B	90.71	0.13
	MOL000388	Gamma-aminobutyric acid	24.09	0.01

**Table 2:** The optimum model for molecular docking.

Small molecule ligand	Receptor protein	Binding energy/kcal.Mol
Baicalein	AKT1	−6.8
Kaempferol	AKT1	−6.1
Quercetin	AKT1	−6
Gamma-aminobutyric acid	IL6	−4.5
Acubin	IL6	−6.8
Paeoniflorin	IL6	−7.8
Quercetin	IL6	−7.8
Aucubin	TNF	−11.8
Kaempferol	TNF	−12.7
Paeoniflorin	TNF	−13
Quercetin	TNF	−9

## Data Availability

The data used to support the findings of this study are included within the article and supplementary information files.

## Supplementary Materials

Supplementary Materials Table S1: all the potential targets of XJDHT. Table S2: known SLE-related targets. Table S3: XJDHT shared 129 intersection targets with known SLE-related targets. Table S4: protein-protein interaction information of XJDHT targets for SLE in STRING 11.0. Table S5: results for GO pathway enrichment analysis. Table S6: results for KEGG pathway enrichment analysis.
